# Prevalence, genotype and antimicrobial resistance of *Clostridium difficile* isolates from healthy pets in Eastern China

**DOI:** 10.1186/s12879-019-3678-z

**Published:** 2019-01-11

**Authors:** Yanxia Wei, Mingchuang Sun, Yuhan Zhang, Jing Gao, Fanyun Kong, Dianbin Liu, Hao Yu, Jinxin Du, Renxian Tang

**Affiliations:** Jiangsu Key Laboratory of Immunity and Metabolism, Laboratory of Infection and Immunity, Department of Pathogenic Biology and Immunology/School of Stomatology, Xuzhou Medical University, Xuzhou, 22104 Jiangsu Province China

**Keywords:** *Clostridium difficile*, MLST, *tcdA*, *tcdB*, Drug resistance

## Abstract

**Background:**

*Clostridium difficile* (*C. difficile*) is a main cause of antibiotic-associated diarrhoea in humans. Several studies have been performed to reveal the prevalence rate of *C. difficile* in cats and dogs. However, little is known about the epidemiology of *C. difficile* in healthy pets in China. This study aimed to assess the burden of *C. difficile* shedding by healthy dogs and cats in China. Furthermore, the genetic diversity and antimicrobial susceptibility patterns of the recovered isolates were determined.

**Methods:**

A total of 175 faecal samples were collected from 146 healthy dogs and 29 cats. *C. difficile* strains were isolated and identified from the feces of these pets. The characterized *C. difficile* strains were typed by multilocus sequence typing (MLST), and the MICs of the isolates were determined against ampicillin, clindamycin, tetracycline, moxifloxacin, chloramphenicol, cefoxitin, metronidazole and vancomycin by the agar dilution method.

**Results:**

Overall, 3 faecal samples (1.7%) were *C. difficile* culture positive. One sample (0.7%) from a dog was *C. difficile* culture positive, while two cats (7.0%) yielded positive cultures*.* The prevalence rate differed significantly between cats and dogs. These isolates were typed into 3 MLST genotypes and were susceptible to chloramphenicol, tetracycline, metronidazole and moxifloxacin and resistant to ampicillin, clindamycin and cefoxitin. Notably, one strain, D141–1, which was resistant to three kinds of antibiotics and carried toxin genes, was recovered in the faeces of a healthy dog.

**Conclusion:**

Our results suggest that common pets may be a source of pathogenic *C. difficile,* indicating that household transmission of *C. difficile* from pets to humans can not be excluded.

## Background

*Clostridium difficile* (*C. difficile*) is a Gram-positive spore-forming anaerobic bacillus thatis a well-known pathogen causing pseudomembranous colitis and antibiotic-associated diarrhoea [1]. *Clostridium difficile* infection (CDI) is also a common cause of enteritis in different animal species [2]. *C. difficile* enterotoxin A (TcdA) and cytotoxin B (TcdB) are mainly responsible for its pathogenesis [3]. In addition, the actin perturbing binary toxin (CDT) was found as an additional toxin in 4–12% of toxigenic *C. difficile.* This additional toxin is composed of two independent components, a catalytic domain (CDTа) and a binding domain (CDTβ) [4]. This toxin has been reported to be independently associated with recurrent CDI [5].

The prevalence of CDI has increased globally due to inappropriate use of antibiotics. Many articles have reported the molecular epidemiology of *C. difficile* isolated from patients in hospitals, which has been studied extensively as an external source of CDI [6]. Additionally, some human pathogenic PCR ribotypes are found in other mammals, such as pigs, horses, and cattle. Food contamination with pathogenic *C. difficile* has been demonstrated in previous reports [7–9]. Although articles have focused on *C. difficile* isolated from animals, these studies have mainly aimed to reveal the possible transmission of *C. difficile* from animal species used for food by humans, such as seafood, beef andpork. [7–10]. However, the zoonotic potential of this pathogen remains controversial.Only a few articles have investigated the molecular epidemiology of *C. difficile* isolated from pets [11–13], and little is known about *C. difficile* in common pets in good health in China. Therefore, this study focused on the epidemiology of *C. difficile* isolated from the most common pets in China, dogs and cats, to potentially indicate another important route of zoonotic transmission of *C. difficile* other than food-borne infection.

This study was performed to assess the burden of *C. difficile* shedding by healthy dogs and cats in Eastern China and reveal the genetic diversity and antimicrobial susceptibility patterns of isolates recovered from these healthy cats and dogs. Three different methods were used to precisely identify *C. difficile* isolates, and multilocus sequence typing (MLST) analysis was performed to type *C. difficile* isolates. To explore whether *C. difficile* carried by pets poses a threat to humanhealth., multiplex PCR was used to detect of toxin genes in *C. difficile*. Additionally, the antimicrobial susceptibility of these *C. difficile* isolates was determined.

## Methods

### Sample collection

Faecal samples were collected from adult pets in pet shops, located in downtown or rural areas of Xuzhou City, Jiangsu Province, China. Xuzhou is located at latitudes of 33°43′~ 34°58’ North and longitudes of 116°22′~ 118°40′ East. The average annual temperature is 14 °C, and the average precipitation is 800 mm. A total of 18 pet shops were included in this study. Solid or semi-solid faecal samples were obtained from individuals of the most popular domestic species of pets that were adult, non-diarrhoeic and clinically healthy. Pets were not included if they had been exposed to antibiotics in the last 3 months before sample collection., A total of 174 faecal samples were collected, including145 samples from dogs and 29 samples from cats. The study was approved by the ethics committee of Xuzhou Medical University. All animal experiments were approved by the Animal Care and Use Committee of Xuzhou Medical University.

### Isolation and identification of *C. difficile*

For enrichment cultivation of *C. difficile*, each faecal sample was introduced into 5 mL of brain heart infusion broth (BHI) (CM1135B, Oxoid) supplemented with 1.0 g/L taurocholic acid sodium salt hydrate (T4009, Sigma) and *C. difficile* selective supplement (SR0096, Oxoid) [8]. After 7 days of incubation at 37 °C in an anaerobic workstation (DG250, Don Whitley Scientific), alcohol-shock was performed by mixing homogenized broth-culture with an equal volume of ethanol (96%) for 50 min at room temperature. After centrifugation, the pellet was collected and spread onto cycloserine cefoxitin fructose agar taurocholate agar plates (CCFAT) [14]. After the plates were incubated anaerobically at 37 °C for 48 h, the presumptive colonies on the plates that demonstrated a typical morphology (flat, irregular yellowish and ground-glass appearance) were selected and subcultured on BHI agar plates with *C. difficile* selective supplement. After 48 h at 37 °C for 48 h, the presumptive isolates were identified by using a *C. difficile* latex agglutination rapid test kit (DR1107, Oxoid) for the detection of *C. difficile* antigen. In addition, the presumptive isolates were subcultured in BHI broth with *C. difficile* selective supplement for 24 h to collect bacterial pellets for DNA extraction and PCR confirmation. For DNA extraction, the cultures were centrifuged at 13400 rpm for 5 min to collect bacterial pellets for DNA extraction using. DNA was extracted from the bacterial pellets according to the protocol provided in the QIAamp DNA Mini Kit (51,304, QIAGEN). Further identification of *C. difficile* was performed by molecular techniques, detection of a species-specific internal fragment of *tpi* by PCR, and sequencing of 16S rDNA as described previously [15, 16]. Previously reported primers targeting *tpi* and 16S rDNA were used to confirm presumptive isolates [15, 16]. The *tpi* forward primer was tpi-F (AAAGAAGCTACTAAGGGTACAAA), and the *tpi* reverse primer was tpi-R (CATAATATTGGGTCTATTCCTAC). PCR-positive isolates were further confirmed by amplification and sequencing of 16S rDNA. The primers for amplification of 16S rDNA were PS13 (GGAGGCAGCAGTGGGGAATA) and PS14 (TGACGGGCGGTGTGTACAAG). All PCRs were performed in an Applied Biosystems thermal cycler (Applied Biosystems 2720, Applied Biosystems) in a final volume of 20 μL/reaction. The reaction mixture consisted of 10 μL of 2╳Taq Plus PCR MasterMix (KT205, TIANGEN), 0.2 μM each primer and 1 μL of template DNA. Unused swabs and tubes (tool and container for sample collecting) were used as the control of lab contamination and included as samples to perform isolation of *C. difficile*, DNA extraction and PCR.

### Multiplex PCR for the detection of toxin genes

A 5-plex PCR was performed to detect the *tcdA*, *tcdB*, *cdtA*, and *cdtB* genes and 16S rDNA [15]. *C. difficile* strain ST1/RT027 (*tcdA*^+^, *tcdB*^+^, *ctdA*^+^, *ctdB*^+^) was used as a positive control for the amplification. The conditions and primers for the PCRs were as previously reported with several modifications [15, 16]. The PCR assay was performed at 94 °C for 10 min, followed by 32 cycles of 94 °C for 50 s, 57 °C for 40 s, and 72 °C for 50 s, and a final extension at 72 °C for 10 min.

### Multilocus sequence typing (MLST) analysis

All of the *C. difficile* isolates were further characterized by MLST. MLST was performed using seven housekeeping genes (*adk*, *atpA*, *dxr*, *glyA*, *recA*, *sodA* and *tpi*) to compare theisolates from pets with human strains [17]. The amplification conditions and oligonucleotide primers for MLST were used as previously reported by Griffiths et al. [17]. Seven PCR products were obtained for each strain and sequenced using PCR forward and reverse primers. The sequences of the allele were submitted to the MLST database homepage. The assignment of the allele numbers, clades and sequence types (STs) wereperformed using the *C. difficile* MLST website (http://pubmlst.org/cdifficile/). The programme MEGA, version 4 (Molecular Evolutionary Genetics Analysis [http://www.megasoftware.net/]), was used to construct a phylogenetic tree by the neighbour-joining method.

### Antimicrobial susceptibility testing

All *C. difficile* isolates were tested for susceptibilities to a total of 8 antimicrobial agents by the agar dilution method according to Clinical and Laboratory Standards Institute (CLSI) guidelines (document M11-A8; CLSI, 2012) [18]. The antimicrobial agents used in this study were ampicillin, clindamycin, tetracycline, moxifloxacin, chloramphenicol, cefoxitin, metronidazole and vancomycin. The breakpoints for antimicrobial agents except vancomycin were determined based on CLSI M100-S27 (CLSI, 2017) as previously reported [19]. For vancomycin, the recommendation of the European Committee on Antimicrobial Susceptibility Testing was used (http://www.eucast.org). The resistance breakpoints for each antimicrobial agent are as follows: ampicillin (*R* ≥ 2 μg/mL), clindamycin (*R* ≥ 8 μg/mL), tetracycline (*R* ≥ 16 μg/mL), moxifloxacin (R ≥ 8 μg/mL), chloramphenicol (*R* ≥ 32 μg/mL), cefoxitin (*R* ≥ 64 μg/mL), metronidazole (*R* ≥ 32 μg/mL) and vancomycin (R ≥ 2 μg/mL). *C. difficile* ATCC 700057 was used for quality control.

### Statistical analysis

Prevalence rates were compared by the χ^2^ test with Yates’ correction. All calculations were performed using Prism 5.0 (GraphPad Software, Inc. USA). A *P*-value < 0.05 was considered statistically significant.

## Results

### Isolation and identification of *C. difficile*

Three of 175 faecal samples analysed were found to contain *C. difficile*. The isolation rate of *C. difficile* was 1.7% for the total faecal samples collected in this study. *C. difficile* was isolated from 2/29 cat faecal samples (7.0%) and 1/146 (0.7%) dog faecal samples The prevalence rates of *C. difficile* in cats and dogs differed significantly, suggesting that *C. difficile* recovery was associated with the pet species.

### Antibiotic susceptibility of *C. difficile* isolates

There is increasing concern about the emergence of multi-drug resistant bacteria among household pets and the possible transmission of resistant strains between pets and their owners. Thus, the susceptibility patterns of the isolates to 8 antibiotics were determined (Table 1). All *C. difficile* isolates characterized in this study were susceptible to chloramphenicol, tetracycline, metronidazole and moxifloxacin. Additionally, isolate C22–3 was resistant to vancomycin, while isolate D141–1 and C23–2 were susceptible. All of the *C. difficile* isolates displayed resistance to the other three antibiotics ampicillin, clindamycin and cefoxitin.

**Table 1:** MICs of 8 antimicrobial agents for 3 *C. difficile* isolated from pets

Antimicrobial agent	Breakpoint of resistant	MIC (μg/ml)		
		D141–1	C23–2	C22–3
Ampicilin	≥2	8	8	8
Chloramphenicol	≥32	<8	<8	<8
Metronidazole	≥32	<0.5	<0.5	0.5
Cefoxitin	≥64	>256	>256	>256
Clindamycin	≥8	>32	>32	>32
Moxifloxacin	≥8	<2	<2	<2
Teracycline	≥16	4	<4	8
Vancomycin	≥2	<0.5	<0.5	>8

### The toxin gene profiles

**Fig. 1:**
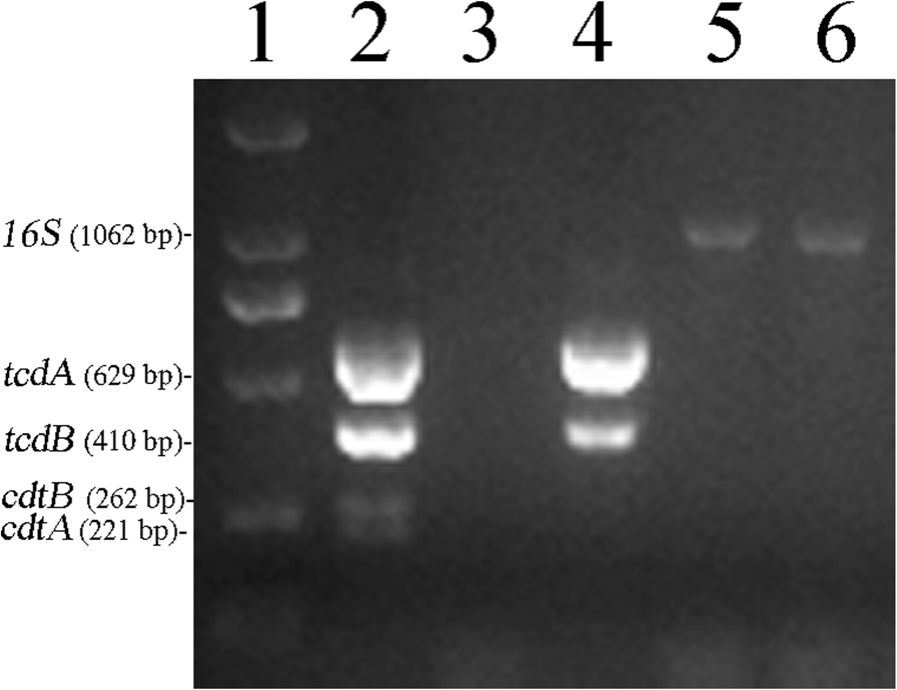
Toxin gene profiles of the three *C. difficile* isolates from pets in this study. Lane 1: DNA marker, D2000; Lane 2: ST1/RT027 (*tcdA*^+^, *tcdB*^+^, *ctdA*^+^, *ctdB*^+^); Lane 3: Negative control; Lane 4: D141–1; Lane 5: C22–3; Lane 6: C23–2. ST1/RT027 (*tcdA*^+^, *tcdB*^+^, *ctdA*^+^, *ctdB*^+^) was used as positive control. The PCR products of 16S DNA, *tcdA*, *tcdB*, *ctdA* and *ctdB* were 1062 bp, 629 bp, 410 bp, 221 bp and 262 bp, respectively

A 5-plex PCR was performed to detectfour *C. difficile* toxin genes *tcdA*, *tcdB*, *cdtA* and *cdtB*. 16S rDNA was used as an internal PCR positive control (Fig. 1). Our results showed that isolate D141–1 contained the toxin genes *tcdA* and *tcdB* but did notcarry the binary toxin genes *cdtA* and *cdtB*. No toxin genes were found in isolate C22–3 and C23–2 isolated from cats, (Table 2). These results suggested the possibility of transmission of toxigenic *C. difficile* from pets to humans via contact.

**Table 2:** Sequence types (STs), allelic profiles and toxin gene profiles

Strains	ST	Clades	Allelic profile^a^	Toxin gene profiles		
				*tcdA*	*tcdB*	*cdtA/cdtB*
C22–3	ST-15	1	1,1,6,1,8,5,1,1	−	−	−/−
C23–2	ST-3	1	1,1,2,1,1,1,1,1	−	−	−/−
D141–1	ST-129	1	1,3,6,1,1,1,3,1	+	+	−/−

^a^The allelic profile is *adk*, *atpA*, *dxr*, *glyA*, *recA*, *sodA* and *tpi* in the order from left to right

### *C. difficile* MLST analysis

**Fig. 2:**
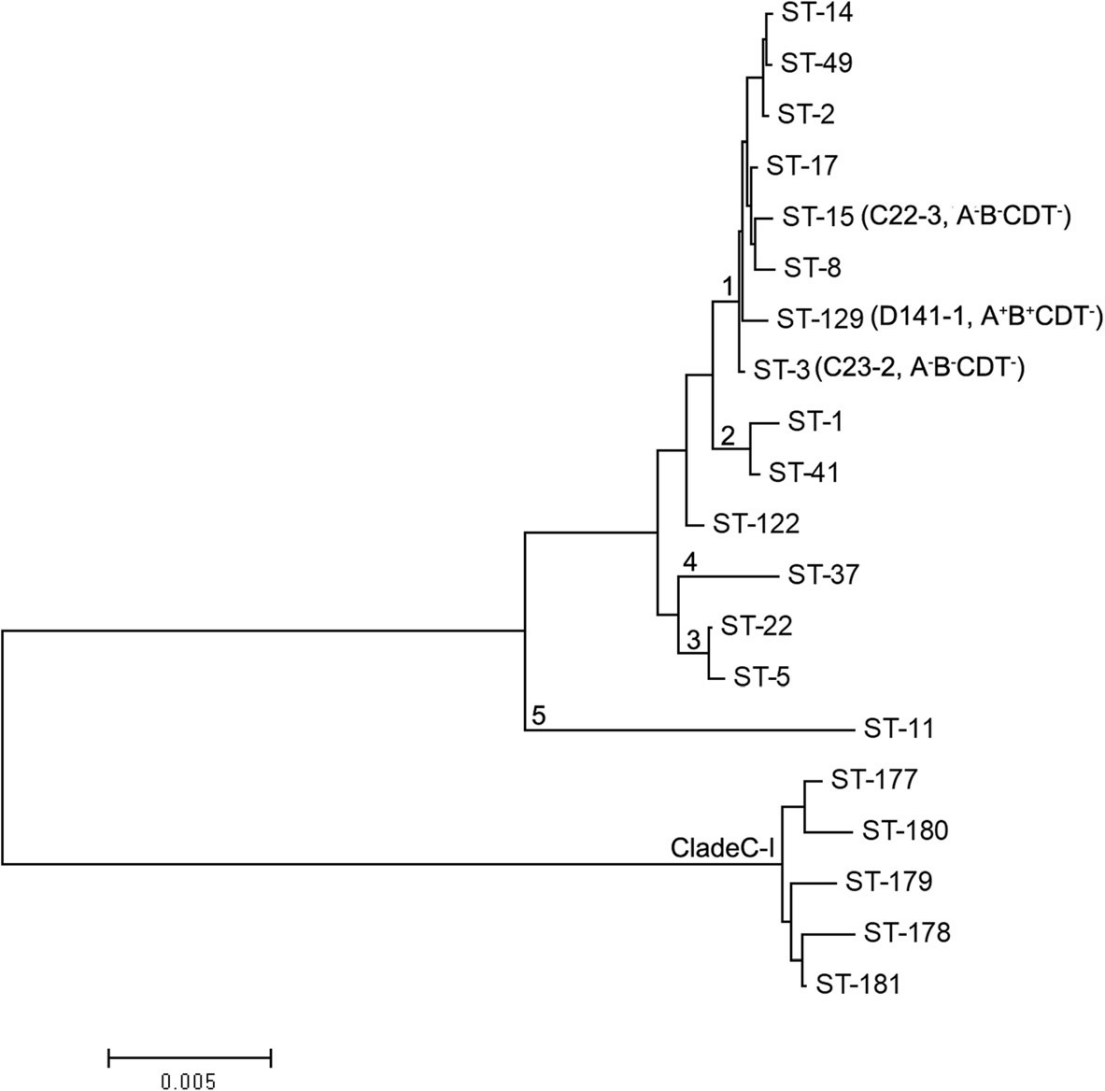
Phylogenetic tree showing the relationships among isolates in this study and the representatives of the six described *C. difficile* clades. Neighbour-joining tree were constructed using the concatenated sequences of the seven loci (3501 nucleotides) used in MLST. The *C. difficile* isolates in this study are indicated by their designated numbers. The STs of well-characterized representatives of each clade are indicated

MLST was performed to further assess the possibility of transmission of *C. difficile* between humans and pets by comparing the diversity of alleles among *C. difficile* strains in this study. All the 3 *C. difficile* isolates were typed by MLST, which showed that the 3 *C. difficile* isolates were assigned to different STs (Table 2). The relationships among the three isolates in this study and between the isolates and other isolates reported previously were examined using phylogenetic analysis based on the sequences of seven housekeeping genes used in MLST as described previously [17]. The results revealed that all three isolates were clustered in Clade 1, ST3, ST15 and ST129 (Fig. 2). Isolates C22–3 and C23–2were assigned to ST-3 and ST-15 (Fig. 2). Isolates D141–1, which was found to carry the toxin genes*tcdA* and *tcdB,* was assigned to ST-129.

## Discussion

Our results revealed that faecal shedding of *C. difficile* is not common among healthy pets in Eastern China. The samples used in this study were collected from 18 pet shops. In pet shops, animals are kept in close contact with each other and may also be exposed to *C. difficile* from their handlers or visitors to the shop. Since the animals were kept in close proximity with each other, the low prevalence found in this study and the fact that no MLST types were shared between animals imply that the transmission of *C. difficile* is not common among animals. These results agree with observations in previous reports on the epidemiology of *C. difficile* in pets from Spanish veterinary teaching hospitals and veterinary clinics in the Madrid region [13, 20]. In the current study, colonization and transient passage of *C. difficile* were not differentiated. In further studies, the repeat collection of stool samples could be performed to reveal whether the isolated *C. difficile* colonize in or transiently pass through pets’ gut. The majority of samples were collected from dogs and the rest were from cats in this study, which may result in a bias of *C. difficile* prevalence in cats. Thus, due to the limited number of samples from cats, the results reported here may not completely represent the prevalence of *C. difficile* in cats in Eastern China. The age of the animal is important for *C. difficile* prevalence, since the pathogen has been isolated more frequently in the faecal samples of juvenile animals. In this study, faecal samples were collected from adult pets. In a future study, faecal samples from juvenile pets could be included, and an analysis of samples from juvenile and adult animals could be performed in a larger survey in China. Notably, one isolate recovered from dog faeces, D141–1, was resistant to three kinds of antibiotics and carried toxin genes (*tcdA* and *tcdB*). Since there is intimate contact between humans and their pets, this result suggests the possibility of transmission of toxigenic *C. difficile* from pets to humans during contact with pets.

Vancomycin and metronidazole have long been used as first-line drugs for the treatment of CDI [21, 22]. In this study, one isolate displayed high-resistance to vancomycin (MIC> 8 μg/ml). All isolates were susceptible to metronidazole (Table 1). A few isolates with low resistance or reduced susceptibility to vancomycin have been were reported in China [23–25]. All *C. difficile* isolates in this study showed resistance to clindamycin and cefoxitin, similar to clinical *C. difficile* in China and other countries [23, 24, 26, 27]. All *C. difficile* isolates in this study exhibited high susceptibility to tetracycline, consistent with clinical isolates in China [23]. Few studies have tested the susceptibility of *C. difficile* to chloramphenicol and ampicilin. The results in this study showed that all three isolates were susceptible to chloramphenicol. Isolate D141–1, which contained toxin genes, showed resistance to 3 different antibiotics, including ampicillin, clindamycin and cefoxitin, which are known to promote CDI [28, 29]. Some studies abroad have reported high resistence of *C. difficile* isolates from food or community patients, with resistance rates of 72.22 and 100%, respectively [28, 30]. Some domestic studies have shown that *C. difficile* isolated from hospitals also displays high resistance to clindamycin (88.1%) and cefoxitin (86.67%) [18, 23].

*C. difficile* can be classified into 5 major clades (Clade 1–5) and 2 novel clades (Clade 6, C-I) using MLST [31]. Clade 1 represents a highly heterogeneous cluster of toxigenic and nontoxigenic STs. This clade includes the majority of human isolate STs, such as ST-2, ST-14, ST-49, ST-8 and ST-17 [17, 31]. Jin et al. and Chen et al. identified more than 30 STs in stool specimens of patients with diarrhoea or patients suspected by clinicians to have CDI in hospitals located in Eastern China. Among them, ST2, ST3, ST35, ST37 and ST54 were the most prevalent types [22, 32]. In the current study, the three isolates were assigned to ST-3, ST-15 and ST129, which have been identified in patients with diarrhoea in Eastern China [22, 32]. These results indicate the potential for *C. difficile* to be transmitted from pets to humans.

Since there is intimate contact between humans and their pets, the isolation of *C. difficile* from pets in this study suggests a possibility that humans may be colonized by *C. difficile* carried by pets, although faecal shedding of pathogenic *C. difficile* was not common among healthy dogs and cats. Given that Clade 1 contains the majority of human isolate STs [17, 31], these results further imply that domesticated pets may be possible community reservoirs of *C. difficile* infection in humans, potentially due to the intimate contact between these pets and their owners.

## Conclusion

There has been a lack of studies of *C. difficile* among animals in China. In this study, *C. difficile* isolates were recovered from the faeces of healthy pets in Eastern China. These results demonstrated that faecal shedding of pathogenic *C. difficile* is not common among healthy dogs and cats in Eastern China. The three isolates were assigned to ST-3, ST-15 and ST129, which have been identified from patients with diarrhoea in Eastern China. Among them, one isolate, D141–1, which contained toxin genes and was genotyped into ST129, was isolated from the faeces of one dog. This result implies a potential association between pets and diarrhoeal infection in humans. In addition, one isolate displaying high resistance to vancomycin was found. In summary, the results of the present study provide evidence that domestic pets may be a reservoir of human pathogenic *C. difficile*, and thus a threat of healthy companion pets to human health by cannot be excluded.

### Nucleotide sequence accession number

The sequences of 16S rDNA of C22–3, C23–2 and D141–1 have been deposited in the GenBank databases with the accession numbers MK246185, MK246184 and MK246131, respectively.

## Abbreviations

*C. difficile*: *Clostridium difficile*
CCFAT: Cycloserine cefoxitin fructose agar taurocholate agar plates
CDI: *C. difficile* infection
CDT: Actin perturbing binary toxin
CDTβ: Binding domain
CDTа: Catalytic domain
CLSI: Clinical and Laboratory Standards Institute
MLST: Multilocus sequence typing
STs: Sequence types
TcdA: Enterotoxin A
TcdB: Cytotoxin B
